# Proline-Mediated Drought Tolerance in the Barley (*Hordeum vulgare* L.) Isogenic Line Is Associated with Lateral Root Growth at the Early Seedling Stage

**DOI:** 10.3390/plants10102177

**Published:** 2021-10-14

**Authors:** Felix Frimpong, Michael Anokye, Carel W. Windt, Ali A. Naz, Michael Frei, Dagmar van Dusschoten, Fabio Fiorani

**Affiliations:** 1 Institute for Bio- and Geosciences, IBG-2: Plant Sciences, Forschungszentrum Jülich GmbH, 52425 Jülich, Germany; c.windt@fz-juelich.de (C.W.W.); d.van.dusschoten@fz-juelich.de (D.v.D.); f.fiorani@fz-juelich.de (F.F.); 2 CSIR-Crops Research Institute of Ghana, Kumasi P.O. Box 3785, Ghana; 3 Center of Life and Food Sciences Weihenstephan, Technische Universität München, 80333 München, Germany; michael.anokye@hhu.de or; 4 Department of Plant Breeding, University of Applied Sciences, 49076 Osnabrück, Germany; a.naz@hs-osnabrueck.de; 5 Institute of Agronomy and Plant Breeding, Justus-Liebig-Universität Gießen, D-35392 Gießen, Germany; Michael.frei@agrar.uni-giessen

**Keywords:** drought, fine roots, lateral roots placement, near-isogenic barley lines, proline, root system architecture

## Abstract

A vigorous root system in barley promotes water uptake from the soil under water-limited conditions. We investigated three spring barley genotypes with varying water stress responses using rhizoboxes at the seedling stage. The genotypes comprised two elite German cultivars, *Barke* and *Scarlett*, and a near-isogenic line, *NIL 143.* The isogenic line harbors the wild allele *pyrroline-5-carboxylate synthase1-P5cs1*. Root growth in rhizoboxes under reduced water availability conditions caused a significant reduction in total root length, rooting depth, root maximum width, and root length density. On average, root growth was reduced by more than 20% due to water stress. Differences in organ proline concentrations were observed for all genotypes, with shoots grown under water stress exhibiting at least a 30% higher concentration than the roots. Drought induced higher leaf and root proline concentrations in *NIL 143* compared with any of the other genotypes. Under reduced water availability conditions, *NIL 143* showed less severe symptoms of drought, higher lateral root length, rooting depth, maximum root width, root length density, and convex hull area compared with *Barke* and *Scarlett*. Within the same comparison, under water stress, *NIL 143* had a higher proportion of lateral roots (+30%), which were also placed at deeper substrate horizons. *NIL 143* had a less negative plant water potential and higher relative leaf water content and stomatal conductance compared with the other genotypes under water stress. Under these conditions, this genotype also maintained an enhanced net photosynthetic rate and exhibited considerable fine root growth (diameter class 0.05–0.35 mm). These results show that water stress induces increased shoot and root proline accumulation in the *NIL 143* barley genotype at the seedling stage and that this effect is associated with increased lateral root growth.

## 1. Introduction

Climate variability and ever more frequent drought events negatively affect cereal production [1,2,3]. Therefore, developing adapted cultivars that maintain yields under reduced water availability is essential [4]. Crop adaptation requires that cultivars adjust their above and below-ground morphology, physiology, and biochemical traits to a specific water stress scenario [4,5]. As a critical below-ground trait, the ability to develop deep roots enables the entire plant to adjust to or avoid reduced water availability [6,7,8]. Changes in root growth and root architecture to short- and long-term drought scenarios are considered possible adaptation strategies that may help stabilize leaf water potential under stress [9]. Essential root traits associated with maintaining plant productivity under drought include small fine root diameters [10], long specific root length, root area, root angle, and considerable root length density, especially within deep soil horizons containing available water [11,12,13].

Under drought conditions, proline plays a role in stabilizing cell membranes, maintaining cell osmotic balance, and preventing electrolyte leakage. Moreover, proline functions as an antioxidant regulating the levels of reactive oxygen species for continual plant growth and development [13]. Biancucci et al. (2015) [14] showed that proline affects the size of the root meristematic zone in Arabidopsis, indicating that proline in this location could modulate and control cell division and differentiation. Proline acts as an osmoprotectant in barley, and its accumulation stabilizes whole-plant photosynthetic performance, growth, metabolism, and grain yield under a water deficit [15,16,17]. Plant proline homeostasis is determined by its biosynthesis, catabolism, and transport. Proline is generally synthesized through the glutamate pathway during osmotic stress. In the glutamate pathway, proline is produced from glutamate by *pyrroline-5-carboxylate* synthetase (*P5CS*) and *pyrroline-5-carboxylate* reductase 1 (*P5CR1*) enzymes [13]. In modern barley (*Hordeum vulgare* ssp. Vulgare), breeding efforts to widen the limited genetic diversity successfully used wild relatives (*Hordeum vulgare* ssp. Spontaneum) as donors of exotic germplasm to enhance cultivated varieties [18]. For instance, through repeated backcrossing of wild barley to recurrent parent *Scarlett*, followed by rounds of selfing and marker-assisted selection, alleles from wild barley, *ISR42-8,* have been introduced into *Scarlett* [19,20]. Muzammil et al. (2018) [21] found that the ancestral allele of *pyrroline-5-carboxylate synthase1* promotes proline accumulation and drought adaptation in cultivated *Scarlett* barley.

Naz et al. (2014) [19] studied barley introgression lines and detected quantitative trait loci (QTLs) that underpin root traits, such as root dry weight, root length, and root volume, all of which promote improved fitness under drought stress. They showed that beneficial alleles underlying the measured root traits originated from wild barley, suggesting the possible use of specific introgressions in cultivated barley from the wild progenitor. These introgression lines bear the wild allele at the *pyrroline-5-carboxylate synthase1* (*P5cs1*) locus [18], which enhances proline accumulation in leaves, leading to a comparatively higher yield under drought [21]. However, the introgression lines’ purity can be improved as they may possess a significant fraction of the wild barley donor genome [22]. Further breeding efforts advanced such introgressions into a more homogeneous near-isogenic type [23]. In our present work, we studied the near-isogenic barley line *NIL 143*, which was generated from several rounds of backcrossing between the drought-tolerant introgression line *S42IL-143* and the cultivar *Scarlett* followed by selfing and aided by marker-assisted selection [24].

So far, few abiotic stress studies have explicitly focused on the contribution of proline to root development in domesticated crops. Drought-inducible proline accumulation in the root apex contributes to 50% osmotic adjustment in this region [25,26]. Through repeated experiments, Shrestha, 2020 [24] demonstrated that the drought recovery rate in *NIL 143* was superior to *Scarlett*. Therefore, we investigated whether proline accumulation contributes to barley lateral root growth under water stress.

For these reasons, our current study investigated the hypothesis that proline accumulation contributes to barley root growth under water stress. We evaluated the root-specific traits of contrasting barley genotypes for proline accumulation in different plant organs and their coping strategies under reduced water availability. To this end, we used a non-invasive root phenotyping tool, soil-filled rhizoboxes, and combined it with root imaging and scanning [27,28]. Soil-filled rhizoboxes make it feasible to evaluate the overall differences among crop varieties and further diagnose specific key below-ground traits that might underlie such variations. Using rhizoboxes, Avramova et al. (2016) [29] demonstrated that phenotypic differences between maize genotypes differing in drought tolerance under field conditions could, to some extent, already be identified at the seedling stage by measurements of root length and shoot biomass. We characterized root architectural traits and root placement (roots positioning within the substrate profile) under water stress and control conditions in barley genotypes, including a breeding line that harbors the wild allele at the *P5cs1* locus. We further assessed whether proline accumulation differs between the roots and shoots of the contrasting barley genotypes and if that led to changes in the net CO_2_ assimilation rate, transpiration rate, plant water potential, leaf chlorophyll content, and roots and shoots morphology.

## 2. Results

### 2.1. Root and Shoot Growth Traits of Barley Lines under Water Stress

We measured barley seedlings’ root and shoot growth traits under well-watered (WW) and water stress (WS) treatments 17 days after the stress onset. Shoot fresh weight (g) differed significantly among treatments but not between genotypes or in the genotype × treatment interaction (Table 1). *Barke* shoot fresh weight (g) of 1.28 was the largest, while 0.9 recorded for *Scarlett* was the smallest under WW (Table 1). Under WS, a marginal increase in shoot fresh weights was detected for *Barke* and *NIL 143* compared to *Scarlett* (Table 1). Shoot dry weight (g) was also significantly different (*p* ≤ 0.001) between treatments but not between genotypes or in terms of interaction (Table 1). The *NIL 143* and *Barke* shoot dry weight of 0.14 were the largest, while 0.10 recorded for *Scarlett* was the smallest genotype under WW (Table 1). Under WS, the *Barke* shoot dry weight (g) of 0.07 was the largest, while 0.05 recorded for *NIL 143* was the smallest (Table 1). Root dry weight (g), on the other hand, significantly differed (*p* ≤ 0.05) between treatments as well as genotypes and in terms of interaction (Table 1). The *NIL 143* root dry weight (g) was the largest at 0.04, while *Scarlett* was the smallest at 0.02, under WW (Table 1). The *Barke* root dry weight (g) of 0.02 was the largest, while *Scarlett* and *NIL 143* were the smallest at 0.01 under WS (Table 1). The root/shoot ratio was significantly different (*p* ≤ 0.001) between treatments but not between genotypes or in terms of interaction (Table 1). Under WW, the *NIL 143* and *Barke* root/shoot ratio were the largest at 0.25, while 0.23 recorded for *Scarlett* was the smallest (Table 1). Under WS, the *Barke* root/shoot ratio was the largest at 0.32, with a larger percentage change variation of 28%, while that of *NIL 143* and *Scarlett* was the smallest at 0.28, compared to WW (Table 1). Except for the treatment effect, no significant genotypic or interaction effect was observed for shoot height (cm), leaf number, and leaf length (cm) under both WW and WS (Table 1). *Barke* had the largest maximum leaf width, 0.7 cm, under WW and WS. Leaf area (cm^2^) was different (*p* ≤ 0.001) between treatments but not genotypes or the interaction (Table 1). *NIL 143* had the largest leaf area, 25.0, while *Scarlett* had the smallest, 16.8, under WW (Table 1). *Barke* leaf area (cm^2^) was the largest, 13.3, while *NIL 143* had the smallest, 8.9, under WS (Table 1).

### 2.2. Root and Shoot Growth Traits of Barley Lines under Water Stress

Chlorophyll-a (FW, µg mL^−1^) under WW was not different among the genotypes (Table 2). Under WS, the Chlorophyll-a content differed significantly among the genotypes and between treatments and the interaction was significant (Table 2). Under WS, *NIL 143* had the highest chlorophyll content of 2.7, while 1.17 recorded for *Scarlett* was the lowest (Table 2). Chlorophyll-a was significantly (*p* ≤ 0.05) positively correlated with stomatal conductance (0.46), % relative leaf water content (0.46), leaf area (0.46), root length density (0.53), and lateral root length (0.48, Figure S2). The plant water potential (Ψ_plant_, MPa) of all genotypes under the WW treatment varied only marginally, and there were no significant differences between the genotypes (Table 2). The Ψ_plant_ of −0.17 recorded for *NIL 143* was the highest, while *Barke* was the lowest at −0.39, under WW. Water stress significantly (*p* < 0.001) decreased the Ψ_plant_ of all the genotypes (Table 2). However, the Ψ_plant_ between the genotypes varied significantly (*p* < 0.05), with *Barke* and *Scarlett* exhibiting the lowest values of −1.2 compared with −0.17 for *NIL 143*, with no significant interaction under WS (Table 2). Significant differences in treatment, genotypes, and interaction effects were found in RWC. WS significantly decreased RWC (*p* < 0.001; Table 2), with *NIL 143* showing higher RWC compared to *Barke* and *Scarlett*. All genotypes maintained RWC above 85% under the WW treatment, with no significant differences (Table 2). Percentage reductions in leaf RWC were minimal for *NIL 143* (41%) compared with 51–53% for *Barke* and *Scarlett* (Table 2). The relative leaf water content correlated significantly and positively (*p* ≤ 0.05) with root length density (0.72), lateral root length (0.70), and total root length (0.66, Figure S2).

The net CO_2_ assimilation rate (µmol m^−2^ s^−1^) under WW was not different among the genotypes (Table 2). Under WS, the net CO_2_ assimilation rate was significantly different between the genotypes and treatments, and the interaction was significant (Table 2). Under WS, *NIL 143* had the highest (19) net CO_2_ assimilation rate, while *Barke* had the lowest (12) and a more considerable percentage change (63%, Table 2). Under WW, stomatal conductance (mol m^−2^ s^−1^) was not different among the genotypes, averaging at 0.5 (Table 2). Under WS, stomatal conductance was significantly different among the genotypes and treatments, and the interaction was significant (Table 2). Under WS, *NIL 143* had the highest (0.18) stomatal conductance, while *Barke* had the lowest (0.09). Stomatal conductance was significantly (*p* ≤ 0.01) positively correlated with RWC (0.86), root length density (0.63), lateral root length (0.83), and total root length (0.73). Under WW, the transpiration rate (mol m^−2^ s^−1^) was not different among the genotypes, averaging at 7.7 × 10^−3^ (Table 2). Under WS, the transpiration rate was significantly different among the genotypes and treatments, and the interaction was significant (Table 2). Under WS, *NIL 143* had the highest (3.7 × 10^−3^) transpiration rate, while *Barke* had the lowest (1.9 × 10^−3^) and a more considerable percentage change (77%, Table 2). Intercellular CO_2_ (Ci, μmol mol^−1^) was not different among the genotypes under WW, averaging at 280.5 (Table 2). Under WS, the Ci was significantly different among the genotypes and treatments, and the interaction was significant (Table 2). Under WS, *NIL 143* had the highest Ci, 208, while *Barke* had the lowest Ci of 172 (Table 2).

### 2.3. Effect of Water Stress on Proline in Barley Seedling Shoots and Roots

**Figure 1:**
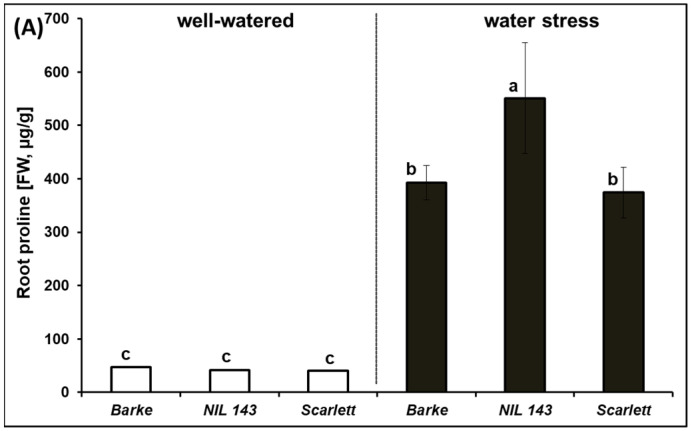

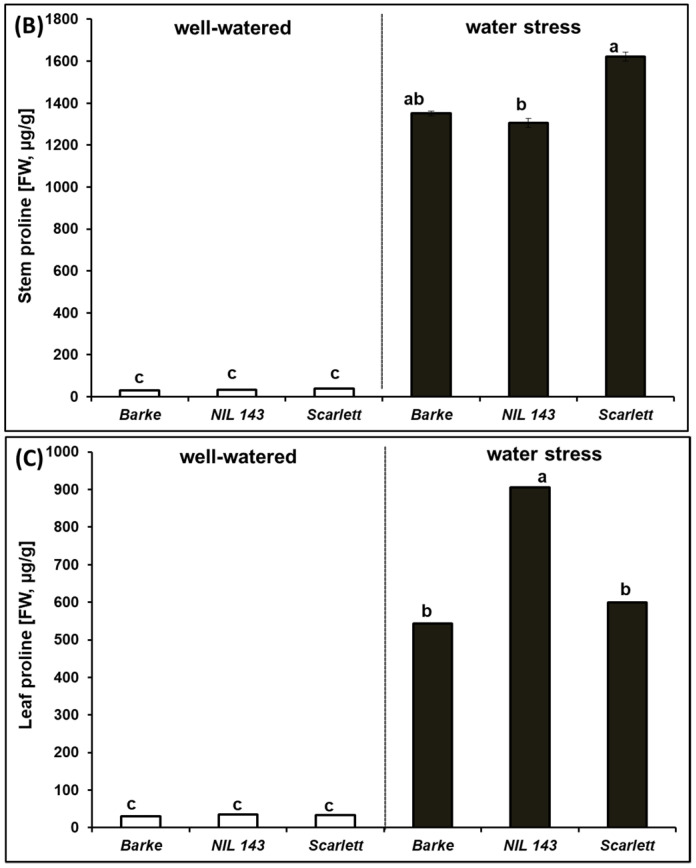
Proline concentration in the root—(**A**); stem—(**B**); and leaf—(**C**) of the barley seedlings 17 days after water stress in rhizoboxes. Significant differences between the genotypes are based on Tukey’s *post hoc* test (α = 0.05) indicated with different letters (n = 6).

We characterized proline accumulation in the leaf, stem, and roots under WW and WS conditions by measuring the concentrations in the shoot (leaf and stem) and root tissues at the seedling stage 17 days after stress. Under WW, the leaf, stem, and root proline concentrations (FW, µg/g) were very low (~35) and not significantly different among all the genotypes (Figure 1). Under WS, the root proline concentration was significantly higher (*p* < 0.001, +40%) for *NIL 143* compared to the elite lines (Figure 1A). Root proline significantly and negatively (*p* ≤ 0.01) correlated with chlorophyll-a (0.47), stomatal conductance (0.64), % relative leaf water content (0.62), leaf area (0.62), root length density (0.60), lateral root length (0.65), and total root length (0.71, Figure S2). Leaf proline was significantly (*p* ≤ 0.05) negatively correlated with plant water potential (0.50, Figure S2). A significant interaction (*p* < 0.01) effect was detected for the leaf and root proline concentrations of the barley seedlings. However, the elite lines accumulated more proline in the stem compared to *NIL 143* under WS, even though no significant differences (*p* > 0.05) were detected (Figure 1B). Significant differences (*p* < 0.001) in treatment but not genotype and no significant interaction effect were found for the stem proline concentration. Quantification of the leaf proline concentration (FW, µg/g) in *NIL 143* together with the elite lines showed a significant increase (*p* < 0.001) in proline accumulation in *NIL 143* up to 906 compared with 600 and 544 for *Scarlett* and *Barke*, respectively, under WS (Figure 1C).

### 2.4. Barley Seedlings’ Root Architectural Traits under Water Stress

**Figure 2:**
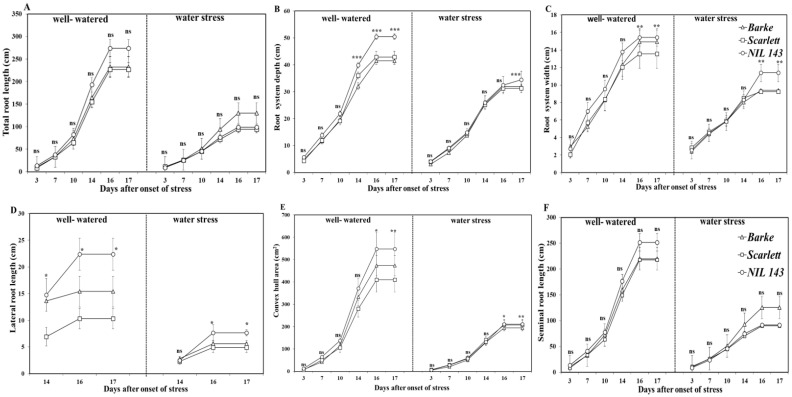
Visible root system growth at the seedling stage over time among the different barley genotypes under well-watered and water stress in rhizoboxes. Plotted are the means fitted with the standard error, n = 6. Significant differences (α = 0.05) among genotypes and treatments at specific days after stress are indicated with asterisks *, **, ***, which follow the standard probability values of 0.05, 0.01, and 0.001, respectively. In the panel: (**A**)—total root length; (**B**)—root system depth; (**C**)—root system width; (**D**)—lateral root length; (**E**)—convex hull area; and (**F**)—seminal root length.

The growth of all the root system traits (total root length, root max width, depth, laterals, seminal roots, and convex hull area) was strongly reduced over time after 14 days of onset of WS (Figure 2A–F). WS significantly decreased (*p* < 0.001, −20%) the length of the visible root system for all the genotypes (Figure 2A–F). Again, for the visible root system traits, we found no significant genotype × treatment interaction (Figure 2). However, we observed genotypic differences in the root system depth, width, lateral root length, and convex hull area, but not in the total and seminal root lengths over time (Figure 2A–F). Under WW, genotypic differences in the lateral roots, depth, and convex hull area were evident early, 14 days after WS start, and lasted until the 17th day of harvest (Figure 2A–F). *NIL 143* exhibited considerable lateral root length relative to the shoots compared to *Barke* and *Scarlett* under WW (Table 1 and Figure 2A,D,F). *NIL 143* had the highest total root length (274 cm), depth (51 cm), width (15 cm), lateral root length (22 cm), convex hull area (548 cm^2^), and seminal root length (252 cm, Figure 2A–F) after 17 days under WW. *Scarlett* had the lowest total (226 cm), lateral (10 cm), and seminal root lengths (225 cm, Figure 2A,D,F) after 17 days under WW.

Under WS, we found significant differences among the genotypes in the maximum root width, depth, lateral root length, and convex hull area but not in the total root length and seminal root length (Figure 2A–F). Under WS, genotypic differences in the lateral roots, width, and convex hull area became evident early, 16 days after WS start, and lasted until harvest (Figure 2A–F). WS *Barke* had the longest total root length (130 cm) and seminal root length (125 cm, Figure 2A,F). WS *NIL 143* had the biggest root system width (34 cm), deeper depth (11 cm), longer laterals (8 cm), and largest convex hull area (211 cm^2^, Figure 2B–E) 17 days after the onset of stress.

**Figure 3:**
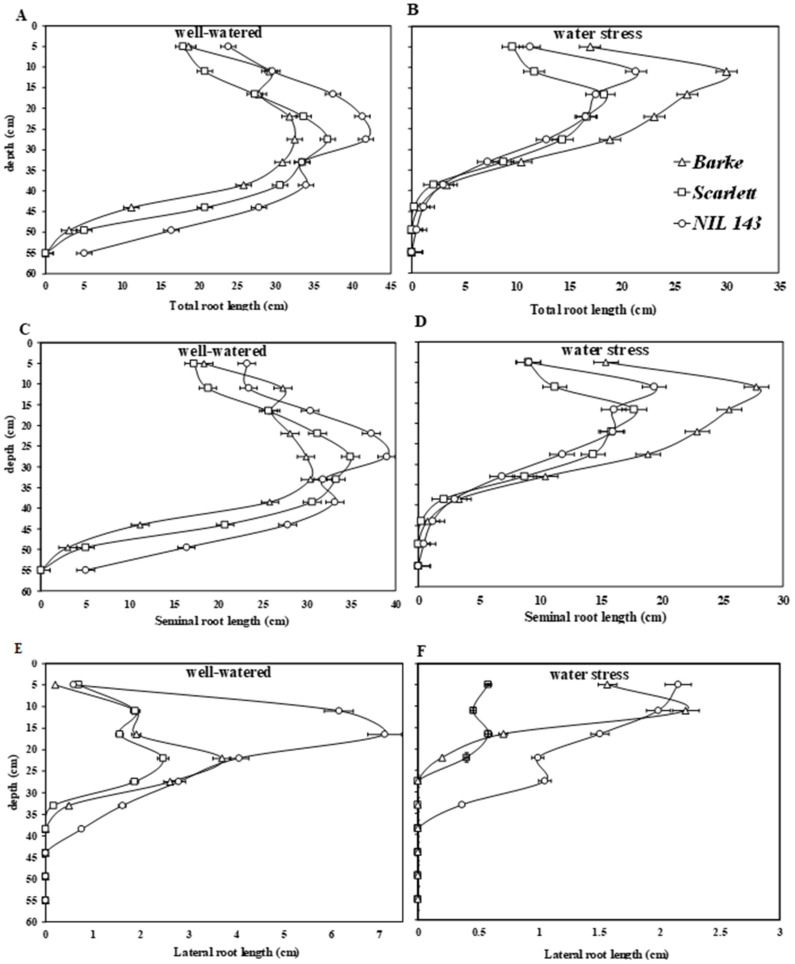
Visible root system placement along the soil profile (0–55 cm) in rhizoboxes for the different genotypes under WW and WS conditions 17 days after the onset of stress. In the panel: (**A**)—visible total root length under well-watered; (**B**)—visible total root length under water stress; (**C**)—visible seminal root length under well-watered; (**D**)—visible seminal root length under water stress conditions; (**E**)—Visible lateral root length under well-watered; and (**F**)—visible lateral root length under water stress conditions. Each point represents root growth averaged among six rhizoboxes per treatment (n = 6). Bars on top are standard errors.

We evaluated the different barley seedlings’ root placement (root positioning within the substrate profile) under limited water conditions. *NIL 143* had the longest and deepest root system, as shown by the total root length, seminal root length, and lateral root length under WW (Figure 3A,C,E). *Scarlett* had the shortest and most shallow lateral roots (Figure 3E), even under WW conditions, compared with the other two genotypes. This trend was, however, different under WS. Under WS, *Barke* had the longest and deepest root system, as shown by total root length and seminal root length values (Figure 3B,D), but not the lateral roots. Under WS, *NIL 143* had significantly deeper and longer lateral roots among the genotypes (+33%, Figure 3F).

**Figure 4:**
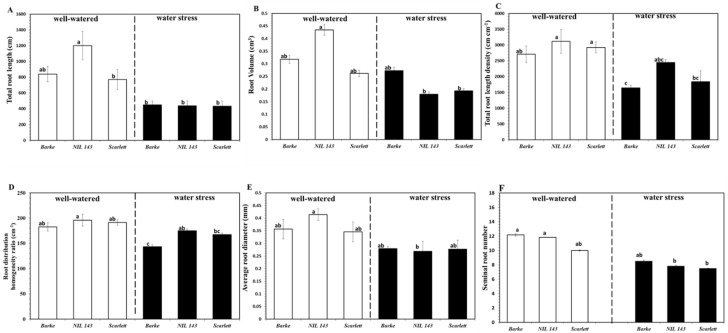
Root architectural traits of the different barley genotypes 17 days after the start of the experiment under well-watered and water stress treatments in rhizoboxes. In the panel are: (**A**)—total root length; (**B**)—root volume; (**C**)—total root length density, (**D**)—root distribution homogeneity ratio; (**E**)—average root diameter; and (**F**)—seminal root number. Plotted are the means and their respective standard error. Different letters on the bars denote significant differences (α = 0.05) based on Tukey’s *post hoc* test for pair-wise comparison, n = 6.

Overall, non-destructive root measurements could estimate approximately 30% of the total root system compared with the root scanned after destructive harvest (Figure 2A and Figure 4A). WinRHIZO scans of the barley seedlings root system 17 days after the onset of WS showed a significant (*p* < 0.001) reduction in the total root length, total root length density, average max root diameter, and seminal root number (−20%, Figure 4A,C,E,F). The root architectural traits analyzed included the total root length (cm), root volume (root diameter × length, cm^3^), total root length density (root length/volume, cm cm^−3^), root distribution homogeneity ratio (root convex hull area/volume, cm^−1^), average diameter (mm), and seminal root number 17 days after the onset of WS (Figure 4A–F). We observed significant genotype × treatment interaction only in the root volume, total root length density, and cumulative fine root length. Under WW, the total root length was not significantly different among the genotypes (Figure 4A). Under WW, *NIL 143* had the highest total root length (1209, Figure 4A). Under WS, *NIL 143* (438)*, Barke* (450)*,* and *Scarlett* (433) recorded no significant differences in the total root length (Figure 4A).

Under WW, root volume (cm^3^) was significantly different among genotypes (Figure 4B). Under WW, *NIL 143* (0.44) had the highest root volume compared to the elite lines (0.30, Figure 4B). We observed genotypic differences in the root volume under WS (Figure 4B). Under WS, *Barke* (0.27) had the highest root volume compared to *NIL 143* (0.18) and *Scarlett* (0.19), respectively (Figure 4B).

**Figure 5:**
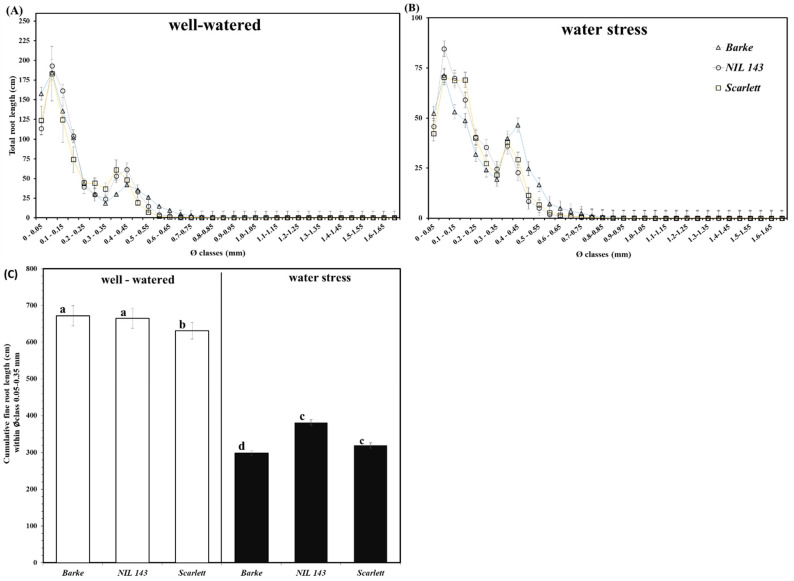
Total root length distribution of all diameter classes of the barley seedlings under WW—(**A**) and WS—(**B**); and cumulative fine root length—(**C**), i.e., the mean sum of the total root length (cm) within seven diameter classes from 0.05 mm up to 0.35 mm) for the different barley genotypes at the seedling stage exposed to well-watered and water stress conditions in rhizoboxes. Different letters on the bars denote significant differences (α = 0.05) based on Tukey’s *post hoc* test for pair-wise comparison, n = 6.

Under WW, the seminal root number was not different among the genotypes (Figure 4F). *Barke* had the highest seminal root number (12), compared to (11) and (10) counted for *NIL 143* and *Scarlett*, respectively (Figure 4F), under WW. We observed no genotypic differences in the seminal root number under WS, with an average of ~8 for all the genotypes (Figure 4F). Under WW, the total root length density (cm cm^−3^) was significantly different among the genotypes (Figure 4C). *NIL 143* had the highest root length density (3064, Figure 4C) under WW. We observed genotypic differences in the root length density under WS (Figure 4C). *NIL 143* had the highest total root length density (2446), while *Barke* had the lowest (1646, Figure 4C) under WS. Under WW, root homogeneity (cm^−1^) was not different among the genotypes (Figure 4D). *Barke* showed the poorest root homogeneity (182, Figure 4D) under WW. Genotypic differences were observed in the distribution of the root homogeneity ratio (cm^−1^) under WS (Figure 4D). *NIL 143* showed a better root homogeneity ratio (175), while *Barke* had the worst (144, Figure 4D) under WS. Under WW, the average max root diameter (mm) was not different among the genotypes (Figure 4D). *NIL 143* had the largest average max root diameter (0.4, Figure 4E) under WW. All genotypes under WS had a similar average max root diameter (~0.3) with no differences (Figure 4E). No differences were observed under both WW and WS in the total root length by diameter distribution between 0 and 1.65 mm (Figure 5A,B). However, in our study, *NIL 143* produced more lateral roots (diameter <0.35 mm) under WS. *NIL 143*’s cumulative fine root length within the first seven diameter classes up to 0.35 mm was higher compared to *Barke* (+22%) and *Scarlett* (+6%) under WS (Figure 5C). Lateral root length was significantly (*p* ≤ 0.001) positively correlated with total root length (0.80, Figure S2).

## 3. Discussion

Several studies reported that variation in fine root structures and deep roots is linked to differences in whole-plant productivity under water limitations [30,31,32]. In the current study, we investigated how proline accumulation relates to barley root growth under water stress. We characterized root system architectural traits and root placement among contrasting barley genotypes, including the breeding line that harbors the wild allele at the *P5cs1* locus under water stress. From our results, the *P5cs1*-isogenic barley line accumulated higher concentrations of root and leaf proline. Further, *NIL 143* had a higher leaf gas exchange, chlorophyll-a content, RWC, root vigor, and less severe dehydration under WS compared with *Barke* and *Scarlett*. The evidence we highlight demonstrates a strong association between organ proline accumulation and lateral root growth under WS in barley at the early seedling stage.

### 3.1. Barley Seedlings’ Root System and Root Placement in Response to Water Stress

We found that *NIL 143* showed less severe symptoms of drought at the shoot level compared with the more severe symptoms exhibited by the other three genotypes. *NIL 143* also showed differences in root system development and placement under reduced water availability. Under WS, *NIL 143* produced longer lateral roots and more lateral roots and placed the roots deeper (+11%) in the substrate compared with *Barke* and *Scarlett* (Figure 3E,F). Compared to the other genotypes, *NIL 143* had a higher proportion of lateral roots (+30%) placed at deeper substrate horizons under WS (Figure 2D, Figure 3F and Figure 5). We also found that *NIL 143* had a comparatively larger root maximum width, root length density, and convex hull area than *Barke* and *Scarlett* under reduced water availability conditions. The wild parental barley accession (*ISR42-8*), from which *NIL 143* was derived, also showed the ability to develop an extensive and deep rooting system [19].

Faye et al. (2019) [32] distinguished drought resistance or tolerance among different pearl millet (*Pennisetum glaucum* L.) based on root length density (total length of roots per unit of soil volume) and the presence of deep lateral roots and fine roots. The reason for choosing this classification is that deeper fine roots and higher root length density define how well plants can take up water and nutrients from available lower layers of the soil [33,34]. Fine roots and root hairs have a larger surface area due to their long combined lengths and are in direct contact with soil water molecules, facilitating water extraction [35,36]. In our study, *NIL 143* had a significantly higher (26%) root length density compared with *Barke* and *Scarlett* (Figure 4C) under WS. This was mainly due to differences in the root growth of specific diameter classes accounting for a larger lateral root of *NIL 143* under WS.

Similarly, Boudiar et al. (2020) [37] reported a remarkable growth (+20%) in lateral roots compared with seminal roots, resulting in better performing modern and landrace barley types grown under low water availability conditions. Under WS, *NIL 143* showed higher root vigor (higher growth of lateral roots, root length density, and fine roots, Figure 2D, Figure 3F and Figure 5C), which likely contribute to capturing water from deeper water soil layers. Han et al. (2016) and Pierret et al. (2016) [38,39] reinforced these suggestions on root vigor (root length density and deep fine roots) of barley seedlings as an important trait under a water deficit. Our data confirm that a more vigorous root system might attenuate the effects of drought at the shoot level.

### 3.2. Organ-Dependent Proline Accumulation in Barley Seedlings Promotes Water Stress Tolerance

We characterized proline accumulation in the leaf, stem, and roots at the seedling stage of the different barley genotypes and how their root traits responded when exposed to WS. To adapt to moisture gradients in the soil, plants alter their physiology, modify root growth and architecture, and exhibit tissue-specific responses [40]. In our study, the genotypes showed varying proline concentrations in the different plant organ tissues under WS (Figure 1A–C). For example, the WS leaf and root tissues (but not the stem tissues) mean the proline concentration was higher in *NIL 143* (+1216 and +650%, respectively) compared to *Barke* and *Scarlett* (Figure 1A–C). *NIL 143* showed a 2-fold higher root and leaf proline concentration and less negative plant water potentials than *Barke* and *Scarlett*. The increased proline concentration by the *NIL 143* in the leaf contributed to less severe dehydration and better turgor and higher performance in the net CO_2_ assimilation rate, stomatal conductance, and transpiration rate compared with *Barke* and *Scarlett* (Table 2 and Figure 1). This agrees well with the findings of Quilambo (2004), Mirza et al. (2019), and Mattioli et al. (2020) [41,42,43], who suggested that proline accumulation in the roots and leaves improves the whole-plant cell membrane integrity and photosynthesis. Generally, the proline concentration was at least 30% higher in the shoot (leaf and stem) than in the roots (Figure 1A–C). This might be caused by the fact that *P5cs1* expression is most highly induced in shoot tissues [44,45].

The root proline concentration under WS was at least 40% higher in *NIL 143* than in *Barke* and *Scarlett* (Figure 1A). Osmotic adjustment due to proline accumulation has been shown to play an essential role in maintaining root elongation at low water potentials [26,46]. A key observation in the peanut nodules (N2-fixing organs in the legume’s root) of the WS-tolerant cultivar (EC-98) was a significant accumulation of osmolytes, including proline [47,48]. However, as an indicator of plants experiencing WS, root proline accumulation negatively correlated with root growth in our study (Figure S2). Earlier reports have interpreted such relationships [48,49] to indicate drought and not the proline effect.

Verslues and Sharp (1999) [50] showed that free proline accumulation in maize roots under a water deficit occurred in the root tips. Under WS, *NIL 143* demonstrated a higher capacity to accumulate proline compared with *Barke* and *Scarlett,* both in the roots and leaves but not in the stem. The above results show that *NIL 143* proline accumulation was targeted at specific plant organs during WS. Bandurska and Stroiński (2003) [51] reported on a resistant wild accession of *Hordeum spontaneum* grown under water-limited conditions associated with its higher constitutive ABA and proline concentrations in the roots and leaves compared with the modern barley cultivar *Maresi*. Their wild *Hordeum spontaneum* genotype further showed a higher capacity to accumulate proline compared with their elite barley, both under mild and severe water deficit conditions [51]. Forde (2014) and Forde et al. (2013) [52,53] attributed their large-scale changes elicited in the root architecture of Arabidopsis mutants to glutamate signaling, a precursor for proline biosynthesis in higher plants. Our data agree with these observations and lend further evidence to the suggestion that drought-inducible proline accumulation in barley is targeted at specific organs, i.e., the roots and leaves.

### 3.3. Proline Led to Changes in the Morpho-Physiological Traits of Barley under Water Stress

It is well established that proline accumulation is a physiological response displayed by several cereal crops exposed to abiotic stresses. Multiple lines of evidence indicate that proline protects cells during osmotic stress by scavenging radical oxygen species; decreasing photo-damage, lipid peroxidation, and buffer redox potential; reducing dehydration; and contributing to signaling for plant defense machinery to come alive [54,55]. In our study, *NIL 143* accumulation of root and leaf proline was associated with the maintenance of leaf gas exchange, higher chlorophyll-a content, less severe dehydration (RWC and Ψ_plant_), and the establishment of deep and long lateral roots under WS. Under WS, *NIL 143* had a higher root proline concentration and more lateral roots and fine roots. In root systems, fine roots and root hairs are the most active portions of the root system in terms of water extraction, with many root tips and intense chemical activity [36]. As a compatible solute, proline accumulation contributes to maintaining the plant cell water potential equilibrium during WS. An increase in proline causes changes in the osmotic potential and cell turgor pressure, promoting the accumulation of potassium and other solutes in the larger cell vacuole [56]. Several other studies indicate that higher leaf and root proline concentrations are associated with greener leaves, cell turgor, cell membrane stability, and improved whole-plant performance in many crop species, including barley [57,58,59,60].

A more than 5-fold increase in spike and leaf proline accumulation occurred in barley introgressions bearing the wild allele of *pyrroline-5-carboxylate synthase1*, which contributed to improved seed yield and whole-plant performance under reduced water availability [60]. In the current study, water stress caused more than a 2-fold increase in the leaf and root proline concentration in *NIL 143* compared with *Barke* and *Scarlett* (Table 1 and Figure 1). The increase in shoot and root proline accumulation was accompanied by higher leaf chlorophyll-*a,* turgor, and photosynthesis in *NIL 143* compared with *Barke* and *Scarlett* (Table 1 and Figure 1). Proline has been implicated in the scavenging of reactive oxygen species that may damage chloroplast membranes under drought [58,61], which might explain the high chlorophyll-*a* content maintained by *NIL 143* under water stress. The drought-inducible *P5cs1* allele from the wild barley introgression into *NIL 143* might have conferred an enhanced higher proline accumulation under water stress. Considerable reductions in chlorophyll content under water stress have been demonstrated in most crop species [62,63]. The decrease in chlorophyll under water stress was mainly caused by active oxygen species damaging chloroplasts [57]. Decreases in the chlorophyll-a content in barley plants under water stress were lower in drought-tolerant than in -susceptible genotypes [14,62,64]. Our observation that the proline accumulators *NIL 143* maintained a high chlorophyll-*a* content under water stress agrees well with these findings.

All the measured physiological traits of all our genotypes were strongly reduced upon WS. Regarding the morpho-physiological shoot differences for the genotypes, *NIL 143* showed a smaller shoot size compared with the roots under WS (Table 1). A higher proline concentration was found in the *NIL 143* root tissues under WS compared with the elite lines. The leaves of *NIL 143* were also greener and showed less negative plant water potential (+35%) and less dehydration (+10%, RWC) than *Barke* and *Scarlett* (Table 2). This genotype thus showed fewer symptoms of drought and improved tolerance to WS.

In contrast to *Barke* and *Scarlett*, under WS, *NIL 143* did not fully close their stomata but were able to keep transpiring and photosynthesizing. An improved RWC and active photosynthesis (optimal rate of net CO_2_, transpiration, stomatal conductance, and other gas exchange parameters) were also reported for related barley breeding lines harboring the same *P5cs1* allele from wild relatives [21,65]. Accordingly, *NIL 143* showed a good ability to replenish and retain water and fewer drought symptoms from the measurements of whole-plant water potential and iWUE under WS (Table 2 and Figure S3A–C). Arguably, the root proline accumulation might have contributed to the improved water retention and turgor of *NIL 143* and resulted in deeper roots, longer lateral roots, and fine root growth under WS (Table 1 and Figure 2, Figure 3 and Figure 4). In relative terms, *NIL 143* showed less pronounced reductions in lateral root growth (−77%) at harvest under WS compared with *Barke* and *Scarlett* (approximately −80%, Figure 2D and Figure 3F). Therefore, our results suggest that under WS, proline accumulation in *NIL 143* contributed to better shoot stomatal conductance, net CO_2_ assimilation, RWC, Ψ_plant_, and root length density compared with *Barke* and *Scarlett*.

We found that the shoot proline (leaf and stem) concentration was 30% higher than the root proline concentration for all genotypes under WS and that these differences were significant. These results indicate that proline accumulates preferentially in developing root systems (Figure 1A–C). Under WS, *NIL 143* had the highest root and leaf proline concentration. *NIL 143* produced the highest growth in lateral roots and fine roots under WS and WW conditions (Figure 2D, Figure 3E,F and Figure 5A–C). Evidence that root growth is stimulated by proline is provided by Biancucci et al. (2015) [14], who reported that exogenous proline stimulated root elongation in Arabidopsis during germination. Under WS, *NIL 143* roots were placed deeper in the soil, indicating a potentially higher ability to take up water from deeper layers. Similar to earlier reports, our results support the evidence that proline accumulation under WS increases cell water stability, promoting growth and metabolism [66,67]. Earlier studies suggested that the cyclic amino acid, proline, has been implicated in root elongation since the discovery of *rolD*, a gene from *Agrobacterium rhizogenes* necessary for hairy roots’ elongation [14,68]. In summary, we found that water stress induces higher shoot and root proline accumulation in specific barley genotypes at the seedling stage and that this effect is associated with pronounced root vigor. We suggest that future studies should explore how proline accumulation might promote root water uptake under drought by acting as an osmolyte.

## 4. Materials and Methods

### 4.1. Growth Condition

All plants were grown in a greenhouse at the Institute of Biosciences and Geosciences (IBG-2), Plant Sciences, Forschungszentrum Jülich GmbH, Germany, August 2020. In the present study, we used two elite German cultivars of malting spring barley (*Hordeum vulgare* L.), ‘*Scarlett*’ and ‘*Barke*’, and a near-isogenic line, *NIL 143*, carrying the wild barley introgression at the *P5cs1* locus derived from the *S42IL-143* genotype [24]. *Barke* was also selected as a negative control having an independent genomic background to compare with *Scarlett* and the progeny. Plants were grown under day/night minimum and maximum temperatures of ~20 ± 2 and 30 ± 2 °C, 16 h during the day and ~19 ± 2 and 20 ± 2 °C 8 h during the night, respectively, at an air humidity of 65 ± 5%. The average vapor pressure deficit inside the greenhouse was approximately 4.7 kPa. Barley seeds for each genotype were pre-germinated on filter paper inside a closed petri dish (between two filter papers imbibed with 1.25 mL of water). Germinated seeds with roots of about 1 cm at one day after sowing were transplanted into rhizoboxes (outer dimensions: 60 × 30 × 3 cm), and manually filled with approximately 6 L of loose sieved black peat soil (Graberde; Plantaflor Humus, Vechta, Germany; containing N, 120 mg L^−1^; P_2_O_5_, 20 mg L^−1^, and K_2_O, 170 mg L^−1^). A 2 cm space was left at the upper open surface of the rhizoboxes to allow subsequent watering. The greenhouse’s daily light integral ranged between a minimum and maximum of 9 and 19 (mol m^−2^ day^−1^), respectively.

### 4.2. Experimental Design

The experiment was a 2 × 3 factorial, randomized complete block design with six replications. There were three genotypes (*Barke, Scarlett,* and *NIL 143*) and two watering regimes (well-watered and water stress) as fixed factors. The soil water content (SWC) of the well-watered (WW) regime was maintained at 70% (g/g). After pre-drying the substrate, SWC of water stress (WS) at the start of the experiment was 40% (g/g) and was further reduced to 6% (g/g) after 17 days. The SWC of both treatments was measured with the aid of a weighing scale, KERN-DBS60-3 (Kern & Sohn GmbH, Balingen, Germany). The estimated soil water potentials (Ψ_soil_) of WW and WS treatments after 17 days were −0.03- and less than −1.51 MPa, respectively. Ψ_soil_ values were estimated using an eight-point water retention curve fitted with the van Genuchten model [69]. Before transplanting seedlings to the rhizoboxes, both WW and WS treatments were supplied with 500 and 200 mL of water, respectively, to enable stand establishment. Subsequently, 60 mL of water were provided three times a week for the WW-treated plants. The WS-treated plants received a one-time watering of 60 mL (BBCH = 12), after which no further watering was given until the experiment was terminated 17 days after sowing. The rhizoboxes’ upper open surface was covered with a 1-cm layer of white plastic beads to prevent water evaporation from the substrate in both treatments. The rhizoboxes were arranged in containers and they were inclined by approximately 45° towards the horizontal plane, with seedlings planted close to the rhizoboxes’ transparent plexiglass view pane, such that root growth could be visualized. A black plastic sheet was used to cover the transparent side plate of the rhizoboxes to prevent light from reaching the roots at all times. The black sheet was only removed briefly for acquiring images (Figure S1).

### 4.3. Root and Shoot Measurements

Shoot height, leaf length, and leaf width were manually measured with a ruler at harvest (17 days after the start of WS treatment). The number of leaves was manually counted at harvest. Leaf area at harvest was determined destructively using an LI-3100C area meter (LI-COR, Lincoln, NE, USA). At harvest, shoot and root fresh and dry weights (g) of plants were determined using a weighing scale XS4002S (Mettler Toledo, Greifensee, Switzerland). Dry weights were measured after samples had been oven-dried at 65 °C for 72 h. Leaf turgid weight was determined after storing fresh leaves overnight in deionized water. The leaf turgid, fresh, and dry weight measurements were used to calculate the percentage relative leaf water content (RWC), [70]. A detailed description of the measured plant traits and the units is shown below (Table S1).

Root measurements were performed using a mobile imaging box for rhizoboxes described by Nagel et al. (2009) [71]. Images of every plant’s root system were manually captured twice every week, starting two days after transplanting. Subsequent photos of the roots were taken until harvest (17 days after sowing). The resulting image sequences were analyzed using the PaintRHIZO software version 3.1 for root growth image analysis by following the protocol developed by Nagel et al. (2009) [71]. The software allows extraction of visible root traits, such as total visible root lengths, seminal root lengths, lateral root lengths, root system depth and width, root surface coverage area, root length density, and root homogeneity distribution along the vertical axis of the rhizoboxes. At harvest, roots were manually washed under running tap water to remove substrate debris. Washed roots were stored in a cold room (10 °C) in Falcon tubes containing 50% ethanol and subsequently scanned at 300 dpi with Epson Expression 12000XL 6.2, Regent Instruments INC., Québec Country, Canada, calibrated for image analysis. The scanned total root system was then analyzed with Regent instrument WinRHIZO^TM^ software, version 2017. The main root traits extracted from the analysis included were total root length (cm), root length distribution per diameter classes (cm^−1^), root volume (cm^3^), root length density (total root length/root volume, cm cm^−3^), average root diameter (mm), seminal root number, and root distribution homogeneity ratio (convex hull area/root volume, cm^−1^).

#### 4.3.1. Gas Exchange and Chlorophyll Fluorescence

We measured six plants per genotype and per treatment, 15 days after WS, using two portable infrared gas analyzers, LI-6800 (LI-COR Inc., Lincoln, NE, USA) with a fluorometer MPF-551065 and MPF-831744, respectively. Measurements were made on fully expanded leaf number four. Light-adapted values included net CO_2_ assimilation (*A*) and stomatal conductance (*gsw*). Measurements were performed with the CO_2_ concentration and temperature in the leaf chamber maintained at 400 µmol mol^−1^ and 25 °C, respectively. The photosynthetic photon flux density was kept at 1500 µmol m^−2^s^−1^ by a red light-emitting diode (LED) light source and at ambient relative humidity of 55% ± 5. All light-adapted parameters were measured between 10:00 a.m. and 12:00 noon to lessen possible variation in parameter values due to diurnal light intensity fluctuations. Intrinsic water use efficiency (iWUE; *A/gsw*) was calculated as the ratio between net CO_2_ assimilation and stomatal conductance. The chlorophyll fluorescence parameter, Fv/Fm, a measurement of the quantum yield of PSII, was performed on fully expanded leaf number four after dark-adaption in a dark room for 45 min. The measurement took place between 20:00 and 21:00. Dark-adapted measurements were performed with the control mode of the LI-6800 set off while the measuring beam was turned on. Multiphase flash was set up as follows: the red target was kept at 8000 µmol m^−2^s^−1^, phases 1 to 3 maintained at 300 ms with a 25% ramp. The output rate and margin were set to 500 Hz and 5 points, respectively.

#### 4.3.2. Plant Water Potential

Plant water potential was determined on fully expanded leaf number four, using the Scholander pressure bomb method [72]. Measurement of the plant water potential was performed between 12:00 noon and 2:00 p.m., when water potential variation is expected to change slowly due to a comparatively higher light intensity. Six plants per genotype per treatment were measured 17 days after WS, BBCH = 15 [73]. Leaves were covered with opaque aluminum foil for about 30 min, which is typically recommended [72], before excision to stop leaf transpiration, allowing the leaf water potential to come into equilibrium with the plant water potential. The entire leaf was detached at the base and wrapped in aluminum foil and sealed inside a pressure chamber (Model 1000 Pressure Chamber, PMS Instrument Company, Albany, SE, USA), leaving the cut end exposed to air. Water appearance at the cut surface of the leaf was observed using a binocular stereo microscope.

#### 4.3.3. Proline Determination

Leaf blades (leaf), leaf sheaths (stem), and root tissues were separated after harvest for proline analysis using six replicates. Samples were quickly placed in plastic vials, closed, and submerged in liquid nitrogen, and later stored in a −80 °C freezer for later use. The stored leaf, stem, and root tissue samples were manually crushed into a fine powder using a ceramic mortar and pestle in liquid nitrogen. The extraction of proline from each tissue was performed by adopting the colorimetric proline determination method described by Bates and Waldren (1973) [74] and Frimpong et al. (2021) [60] with slight modifications. Acid-ninhydrin was first prepared by warming 2.5 g of ninhydrin in 60 mL of glacial acetic acid and 40 mL of 6 M phosphoric acid, with vigorous agitation using a magnetic stirrer until completely dissolved. The solution was covered with aluminum foil to avoid exposure to light and stored in a 4 °C refrigerator for 24 h before use. Then, 100 mg of the crushed tissue samples were weighed into chilled 2 mL Eppendorf tubes and homogenized in 1.5 mL of 3% sulfosalicylic acid by vortexing. The mixture was centrifuged at 12,000 rpm for 10 min. After centrifugation, 500 µL of the sample extract (supernatant) were mixed with 500 µL of glacial acetic acid and 500 µL of ninhydrin reagent in glass tubes (fitted with lids). The mixture was then vigorously vortexed, and incubated at 95–100 °C for 45–60 min in an HB-1000 Hybridizer oven (UVP, Inc., Cambridge, UK). The reaction was terminated quickly with ice. The reaction mixture was extracted with 1.5 mL of toluene, and mixed vigorously by vortexing. The solution was left at room temperature for 30 min to settle until the two phases separated. Then, 100 µL of the chromophore (upper phase) were carefully pipetted into 96-well plates and read with a microplate reader (Synergy™ 2 Multi-Mode, BioTek, Winooski, VT, USA). An empirical calibration curve based on eight points of proline standard concentrations (0, 10, 20, 30, 50, 70, 90, and 100 µg/g) yielded a linear regression of r^2^ = 0.99 between the proline concentration and the measured absorbance at 520 nm, which was used to determine the proline concentrations in the samples.

#### 4.3.4. Chlorophyll Determination

Chlorophyll-a was determined after harvesting six replicates of whole leaves of fully expanded leaf number four of each genotype between 9:00 a.m. and 10:00 a.m. CET using the protocol by Markwell et al. (1986) [75] with slight modifications. First, 40 mg of the crushed leaf tissues were weighed into 2 mL Eppendorf tubes with two metal balls pre-cooled in liquid nitrogen. From here, the reaction was cooled on ice, 1 mL of 95% ethanol (EtOH) plus 0.5 g of CaCO_3_ were added, and the samples were extracted by milling and homogenizing twice (1 min intervals) in pre-cooled Eppendorf-racks using Retsch tissue lyser MM400 (Mixer mill 400, Available online: www.retsch.com, accessed on 25 January 2021). Samples were then centrifuged for 15 min at 4 °C at 12,000 rpm. After centrifugation, the supernatant was carefully transferred into new 2 mL Eppendorf tubes on ice. The extraction was repeated by adding 1 mL of EtOH + CaCO_3_ to the pellet, milled twice in the pre-cooled racks, centrifuged, and supernatants combined. Then, 200 µL of supernatant were diluted with 800 µL of EtOH + CaCO_3_ and mixed by inverting. Three portions of 150 µL of each sample and a blank (EtOH + CaCO_3_) were pipetted into a 96-well plate on ice and absorbance measured at 470, 649, and 664 nm using the microplate reader (Synergy™ 2 Multi-Mode, BioTek, Winooski, VT, USA). The calculation for chlorophyll a was done using the equation below [76]:

(1) $$\mathrm{Chlorophyll}-a\;[µg/\mathrm{mL}]=\frac{13.36\times A664-5.19\times A649}{weight\;of\;sample\times 10\times 1/0.45}$$

*A*649/664 = absorbance at 649 and 664 nm, respectively, and a factor of 10 was used to account for dilution.

### 4.4. Statistical Analysis

All data satisfied the normality and homogeneity test (Shapiro-Wilk and Levene’s test, *p* > 0.05, respectively). Data were then subjected to a generalized linear model (2) analysis:

(2) $$\mu_{ijk}=\mu +\alpha_{i}+\beta_{j}+(\alpha \beta_{ij})+\varepsilon_{ijk},$$

where μ = mean, $\alpha_{i}\;and\;\beta_{j}$ = main effects of water stress treatment and genotypes of the ith and jth levels, $\left(\alpha \beta_{ij}\right)$ represents the interaction effect, and $\varepsilon_{ijk}$ is the error term of the two-way analysis of variance (ANOVA) using the “Agricolae package” built-in ‘R’ statistical software 4.0 [77]. A *post hoc* Tukey test (α ≤ 0.05) was performed to compare the treatment means (Supplementary Materials Datasheet). The Spearman correlation coefficient for pair-wise comparison was calculated for selected plant traits.

## 5. Conclusions

The total root system of all genotypes under water stress at the seedling stage was considerably reduced (−20%) relative to well-watered plants. We observed varying organ proline concentrations for all genotypes as it increased by more than 30% in the shoot compared to the roots under WS. *NIL 143* accumulated higher root, leaf, and not stem proline and showed a comparatively better net CO_2_ assimilation, transpiration, stomatal conductance, plant water potential, and RWC compared with *Barke* and *Scarlett. NIL 143* reduced its seminal roots but increased fine and lateral roots (+30%), improving tolerance under reduced water conditions at the seedling stage. Root growth was therefore enhanced in *NIL 143* because it could maintain its water status under WS. The results suggest that water stress may induce higher shoot or root proline accumulation in *NIL 143* at the seedling stage to stimulate fine or lateral root growth. Future studies should explore the variations in root-shoot growth observed for *NIL 143* in the field to test its performance under a water-limited environment. Further studies will be required to explore how proline accumulation promotes barley root water uptake under water stress.

## Figures and Tables

**Table 1:** Root and shoot traits among the different barley genotypes 17 days after the onset of water stress in the rhizoboxes experiment.

Trait		Treatment			
	Genotype	WW	WS	*p*-Values	% Change
Shoot height (cm)	*Barke*	22.8 ± 1.8 ^ab^	16.3 ± 1.3 ^c^	Genotype ^NS^	−29
	*Scarlett*	20.8 ± 0.9 ^abc^	17.6 ± 1.3 ^bc^	Treatment ***	−9
	*NIL 143*	25.8 ± 1.6 ^a^	16.2 ± 1.6 ^c^	G × T ^NS^	−30
Leaf number	*Barke*	3.2 ± 0.2 ^ab^	2.5 ± 0.2 ^b^	Genotype ^NS^	−21
	*Scarlett*	3.5 ± 0.2 ^ab^	2.7 ± 0.2 ^b^	Treatment ***	−24
	*NIL 143*	4.0 ± 0.3 ^a^	2.8 ± 0.3 ^b^	G × T ^NS^	−30
Leaf length (cm)	*Barke*	11.4 ± 0.9 ^a^	8.8 ± 0.3 ^ab^	Genotype ^NS^	−23
	*Scarlett*	9.4 ± 1.0 ^ab^	9.35 ± 0.4 ^ab^	Treatment ^NS^	−5
	*NIL 143*	8.8 ± 1.7 ^ab^	8.8 ± 0.9 ^b^	G × T ^NS^	−27
Max-Leaf width (cm)	*Barke*	0.73 ± 0.1 ^a^	0.66 ± 0.01 ^bc^	Genotype ***	−9
	*Scarlett*	0.57 ± 0.0 ^ab^	0.52 ± 0.02 ^c^	Treatment *	−10
	*NIL 143*	0.62 ± 0.0 ^abc^	0.53 ± 0.03 ^c^	G × T ^NS^	−16
Leaf area (cm^2^)	*Barke*	23.9 ± 2.5 ^a^	13.3 ± 2.8 ^bc^	Genotype ^NS^	−45
	*Scarlett*	16.8 ± 2.5 ^ab^	11.1 ± 2.5 ^c^	Treatment ***	−34
	*NIL 143*	25.0 ± 2.5 ^a^	8.9 ± 2.5 ^c^	G × T ^NS^	−64
SFW (g)	*Barke*	1.28 ± 0.1 ^a^	0.48 ± 0.1 ^b^	Genotype ^NS^	−60
	*Scarlett*	0.90 ± 0.1 ^a^	0.45 ± 0.1 ^b^	Treatment ***	−46
	*NIL 143*	1.33 ± 0.2 ^ab^	0.48 ± 0.1 ^b^	G × T ^NS^	−57
SDW (g)	*Barke*	0.14 ± 0.01 ^a^	0.07 ± 0.0 ^bc^	Genotype ^NS^	−49
	*Scarlett*	0.10 ± 0.01 ^ab^	0.06 ± 0.0 ^bc^	Treatment ***	−40
	*NIL 143*	0.14 ± 0.02 ^a^	0.05 ± 0.0 ^c^	G × T ^NS^	−60
RDW (g)	*Barke*	0.035 ± 0.002 ^ab^	0.020 ± 0.01 ^c^	Genotype **	−75
	*Scarlett*	0.023 ± 0.002 ^c^	0.018 ± 0.01 ^c^	Treatment ***	−28
	*NIL 143*	0.041 ± 0.002 ^a^	0.018 ± 0.01 ^c^	G × T *	−128
Root/Shoot ratio	*Barke*	0.25 ± 0.021 ^ab^	0.32 ± 0.02 ^a^	Genotype ^NS^	+28
	*Scarlett*	0.22 ± 0.021 ^b^	0.28 ± 0.02 ^ab^	Treatment ***	+27
	*NIL 143*	0.25 ± 0.01 ^ab^	0.28 ± 0.003 ^ab^	G × T ^NS^	+12

SFW: shoot fresh weight. SDW: shoot dry weight. RDW: root dry weight. G × T: genotype × treatment interaction, NS: No significance. Data are means ± standard error (n = 6) after the two-way ANOVA. Significant differences between the genotypes based on the Tukey test (α = 0.05) are indicated with different letters. Asterisks: *, **, *** follows the standard probability values of 0.05, 0.01, and 0.001, respectively. Genotypes were compared within and between treatments. The % change was calculated as the mean difference between drought and control conditions expressed as a percentage.

**Table 2:** Physiological plant traits of different barley genotypes 17 days after the onset of water stress in the rhizoboxes experiment.

Parameter		Treatment			
	Genotype	WW	WS	*p*-Values	% Change
Chlorophyll-a (FW, µgmL^−1^)	*Barke*	2.37 ± 0.3 ^a^	1.07 ± 0.1 ^b^	Genotype ***	−24
	*Scarlett*	2.14 ± 0.0 ^a^	1.17 ± 0.0 ^b^	Treatment ***	−19
	*NIL 143*	2.89 ± 0.2 ^a^	2.69 ± 0.0 ^a^	G × T *	−7
Fv/Fm	*Barke*	0.8 ± 0.0 ^a^	0.81 ± 0.0 ^a^	Genotype ^NS^	0
	*Scarlett*	0.81 ± 0.0 ^a^	0.81 ± 0.0 ^a^	Treatment ^NS^	0
	*NIL 143*	0.81 ± 0.0 ^a^	0.81 ± 0.0 ^a^	G × T ^NS^	0
*A* (µmol m^−2^ s^−1^)	*Barke*	32.3 ± 0.3 ^a^	12.0 ± 0.6 ^d^	Genotype ***	−63
	*Scarlett*	27.5 ± 1.9 ^b^	14.4 ± 0.3 ^d^	Treatment ***	−48
	*NIL 143*	30.4 ± 0.9 ^ab^	19.4 ± 0.4 ^c^	G × T ***	−36
*gsw* (mol m^−2^ s^−1^)	*Barke*	0.602 ± 0.06 ^a^	0.086 ± 0.01 ^c^	Genotype *	−86
	*Scarlett*	0.533 ± 0.02 ^a^	0.105 ± 0.02 ^c^	Treatment ***	−80
	*NIL 143*	0.530 ± 0.02 ^a^	0.183 ± 0.02 ^b^	G × T ***	−65
*E* (mol m^−2^ s^−1^)	*Barke*	8.2 × 10^−3^ ± 1.7 × 10^−4 a^	1.9 × 10^−3^ ± 2.3 × 10^−4 c^	Genotype **	−77
	*Scarlett*	7.2 × 10^−3^ ± 1.5 × 10^−4 a^	2.2 × 10^−3^ ± 4.9 × 10^−4 c^	Treatment ***	−69
	*NIL 143*	7.8 × 10^−3^ ± 2.3 × 10^−4 a^	3.7 × 10^−3^ ± 1.7 × 10^−4 b^	G × T ***	−53
iWUE (µmol CO_2_ mol^−1^ H_2_O)	*Barke*	52.6 ± 3.14 ^c^	134.41 ± 3.14 ^a^	Genotype **	−155
	*Scarlett*	54.5 ± 3.14 ^c^	122.5 ± 3.14 ^a^	Treatment ***	−125
	*NIL 143*	56.4 ± 3.44 ^c^	104.2 ± 3.14 ^b^	G × T ***	−85
Ci (μmol mol^−1^)	*Barke*	277.9 ± 3.3 ^a^	171.7 ± 7.1 ^c^	Genotype ***	−178
	*Scarlett*	284.6 ± 5.1 ^a^	183.4 ± 7.7 ^bc^	Treatment *	−185
	*NIL 143*	279.0 ± 2.9 ^a^	208.0 ± 6.9 ^b^	G × T *	−179
Plant water potential, Ψ (Mpa)	*Barke*	−0.394 ± 0.2 ^ab^	−1.23 ± 0.11 ^c^	Genotype **	−75
	*Scarlett*	−0.293 ± 0.2 ^ab^	−1.26 ± 0.11 ^c^	Treatment ***	−121
	*NIL 143*	−0.170 ± 0.2 ^a^	−0.17 ± 0.11 ^bc^	G × T ^NS^	−78
% RWC	*Barke*	98 ± 2.0 ^a^	46 ± 1.8 ^c^	Genotype *	−51
	*Scarlett*	88 ± 2.0 ^ab^	41 ± 1.8 ^c^	Treatment ***	−53
	*NIL 143*	95 ± 2.2 ^a^	59 ± 1.7 ^b^	G × T *	−41

Fv/Fm: maximum quantum efficiency of PSII, *A*: Net CO_2_ assimilation, *E:* transpiration, *gsw:* stomatal conductance, iWUE (A/gsw): intrinsic water use efficiency, Ci: Intercellular CO_2_, % RWC: percentage relative leaf water content, G × T: genotype × treatment interaction, FW: fresh weight. NS: no significance. Data are means ± standard error (n = 6) after the two-way ANOVA. Significant differences between the genotypes based on Tukey’s *post hoc* test (α = 0.05) are indicated with different letters. Asterisks: *, **, *** follows the standard probability values of 0.05, 0.01, and 0.001, respectively. The % change was calculated as the mean difference between drought and control conditions expressed as a percentage.

## Data Availability

The raw datasets are available upon request to the corresponding author/authors. All additional data in the study are included in the article/Supplementary Materials. Further inquiries can be directed to the corresponding author.

## Supplementary Materials

The following are available online at https://www.mdpi.com/article/10.3390/plants10102177/s1, Figure S1: Experimental setup of barley seedlings in rhizoboxes inclined at 45 °C at the greenhouse (**A**) and a pictorial illustration of the root system as affected under well-watered (**B**) and water stress (**C**) conditions 17 days after treatment application, Figure S2: Trait relationships according to the Spearman correlation coefficient of measured roots, shoots and physiological parameters. Significant correlations “*, **, ***” follows the standard probability values (*p* ≤ 0.05, *p* ≤ 0.01 or *p* ≤ 0.001), Figure S3: Greenhouse pot (1.5 L) experiment comparing the barley near-isogenic line, *NIL 143* and the two elite lines, *Scarlett* and *Barke,* under 14 days continuous soil drying conditions. Soil water content (SWC, (**A**)) and water use (**B**) were recorded twice a week until harvesting. Final shoot dry weight was measured at the end of the experiment, and whole-plant water use efficiency (WUEplant, (**C**)) was calculated as the ratio between final shoot dry weight and water use. Data are means ± standard error (n = 3), Table S1: Summary of shoot and root morphometrics, their description, and units.
