# Supplementary material for: Proline-Mediated Drought Tolerance in the Barley (Hordeum vulgare L.) Isogenic Line Is Associated with Lateral Root Growth at the Early Seedling Stage
Source: Plants (Basel). 2021 Oct 14;10(10):2177. doi: 10.3390/plants10102177 (PMC8538694; doi:10.3390/plants10102177)
Supplement: Supplementary file 1 [file plants-10-02177-s001.zip › plants-1414923-supplementary.pdf]

Table S1. Summary of shoot and root morphometrics, their description, and units.

Plant trait	Description	Unit
**Number of leaves (NL)**	The number of leaves/plants were counted at harvest	count
**Leaf area (LA)**	Destructive using LI-3100C Area Meter (LI-COR, Lincoln, NE, USA) at harvest	cm^2
**Light and dark instantaneous leaf-level gas exchange**	*A, gsw, E, iWUE(A/gsw) Fv/Fm* (Licor 6800)	µmol $mol^{-1}s^{-2}$
**Shoot fresh weight (SFW)**	Plants were cut and weighed individually	g
**Shoot dry weight (SDW)**	Plants were dried in the oven at 65º C for 72 h and weighed	g
**Root fresh and dry weight (RFW, RDW)**	Root fresh and dry biomass were cut and weighed before and after oven drying (65 º C for 72 h), respectively.	g
**Visible root architectural traits**	The visible root traits per plant were analyzed with PaintRHIZO software by following the protocol developed by (Nagel et al., 2009)	cm
**Root architectural traits**	Total root system length and diameter were scanned with WinRHIZO after harvest	cm
**% Relative leaf water content (RWC)**	$\mathbf{RWC} = \frac{fresh\ weight - dry\ weight}{turgid\ weight - dry\ weight} \times \mathbf{100}$ (Tahara et al., 1990)	%

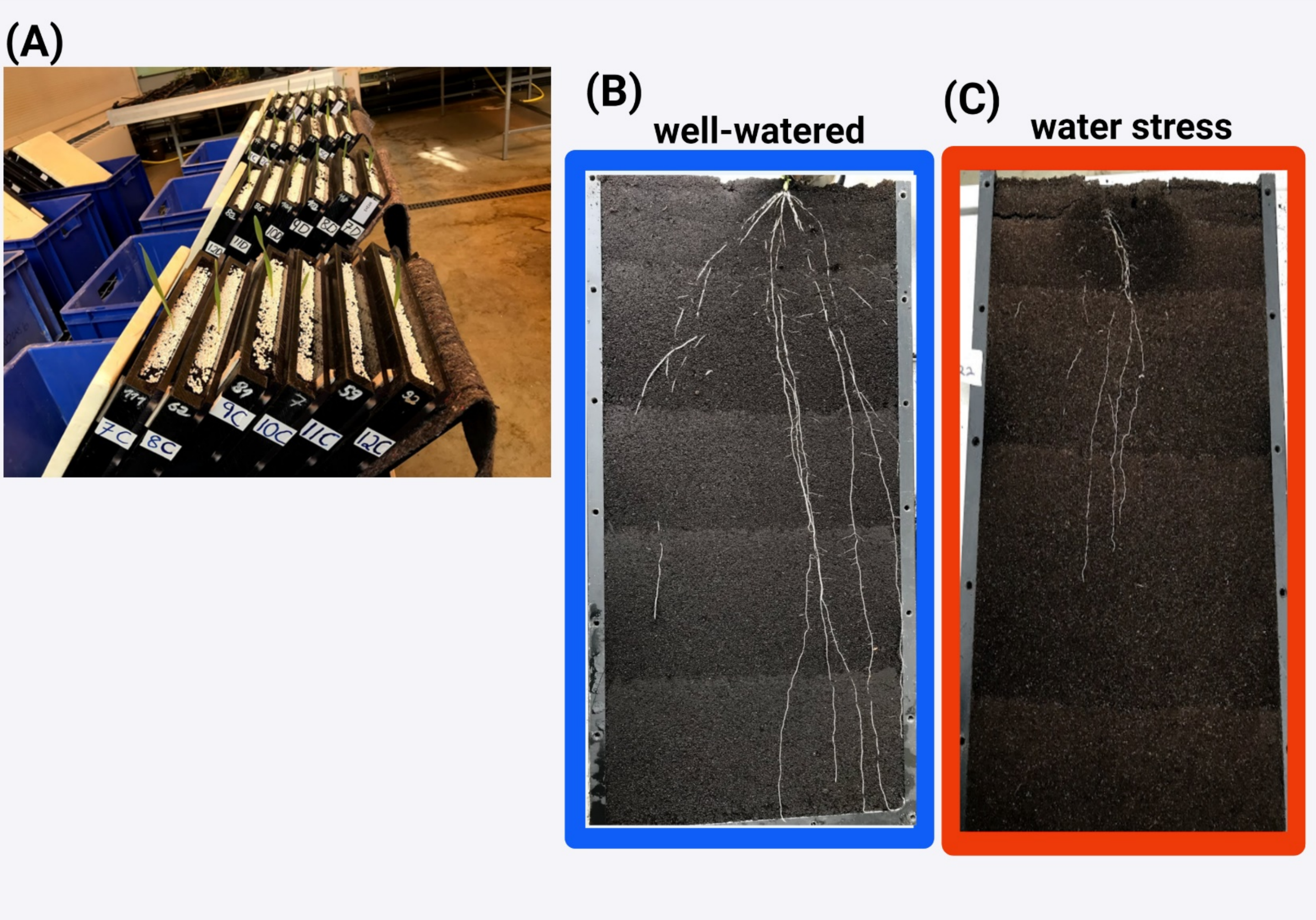

Supplemental Figure S1. Experimental setup of barley seedlings in rhizoboxes inclined at 45˚C at the greenhouse (A) and a pictorial illustration of the root system as affected under well-watered (B) and water stress (C) conditions 17 days after treatment application.

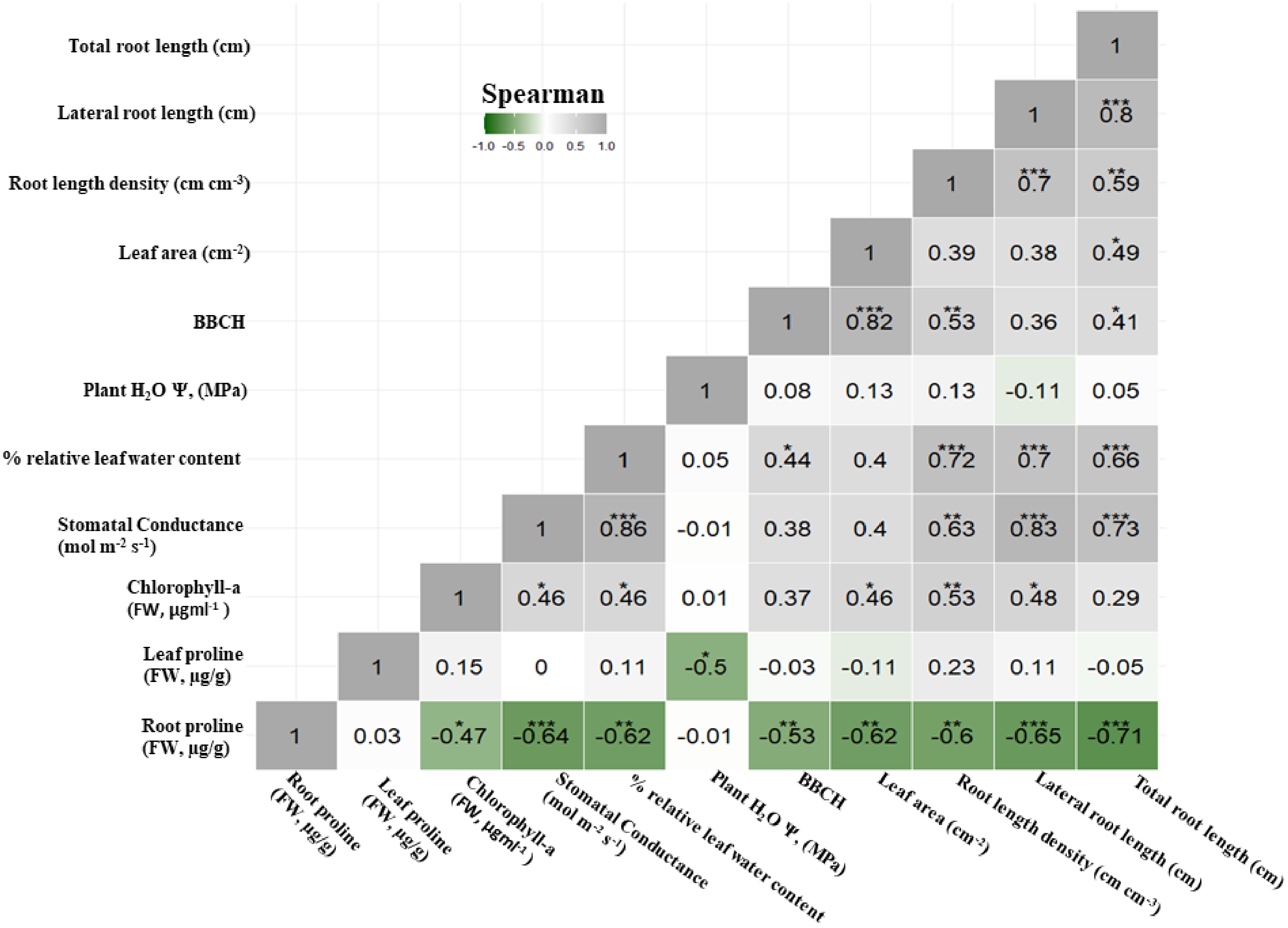

Supplemental Figure S2. Trait relationships according to the Spearman correlation coefficient of measured roots, shoots and physiological parameters. Significant correlations "*, **, ***" follows the standard probability values ($P \leq 0.05$, $P \leq 0.01$or $P \leq 0.001$).

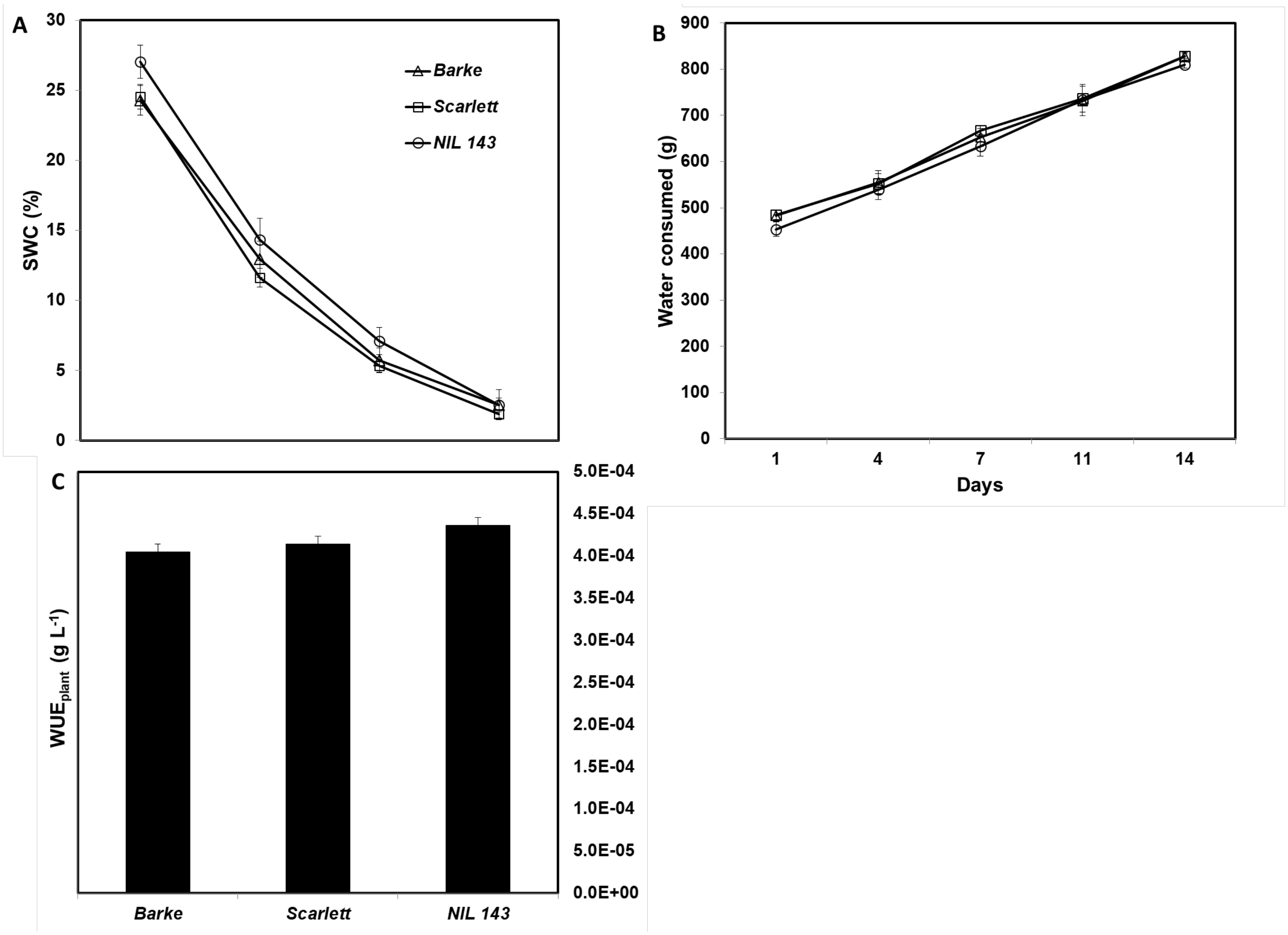

Supplemental Figure S3. Greenhouse pot (1.5 L) experiment comparing the barley near-isogenic line, *NIL 143* and the two elite lines, *Scarlett* and *Barke,* under 14 days continuous soil drying conditions. Soil water content (SWC, A) and water use (B) were recorded twice a week until harvesting. Final shoot dry weight was measured at the end of the experiment, and whole-plant water use efficiency (WUEplant, C) was calculated as the ratio between final shoot dry weight and water use. Data are means ± standard error (n=3).
